# 

**Published:** 1908-10-03

**Authors:** 


### BOOKS RECEIVED.

Ash and Co., Ltd., Southwark.

"A Handbook of Sanitary Law for the Use of Candidates for Public Health Qualifications." By B. Burnett Ham, D.P.H., M.D.

### THE GRADUATES' MEDICAL SCHOOLS.

DIARY FOR WEEK OCT. 5 TO OCT. 10.

**ST. JOHN'S HOSPITAL FOR DISEASES OF THE SKIN, Leicester Square, W.C.**

**At 6 p.m.**

**Oct. 8,** Dr. M. Dockrell, **Cataphoresis in General and Special Medicine.**

**MEDICAL GRADUATES' COLLEGE AND POLYCLINIC, 22 Chenies Street, W.C.**

**At 5.15 p.m.**

**Oct. 5,** Mr. Peter Daniel, **The Treatment of Surgical Tuberculosis by Iodoform, Glycerine, and Bier's Congestion Method.**

**Oct. 6 and 7,** Mr. A. H. Cheatle, **The Surgical Anatomy of the Temporal Bone.**

**Oct. 8,** Mr. Stephen Mayou, **Ophthalmia Neonatorum.**

**NORTH-EAST LONDON POST-GRADUATE COLLEGE, Prince of Wales's General Hospital, Tottenham, N.**

**At 4.30 p.m.**

**Oct. 8,** Mr. Jonathan Hutchinson, F.R.S., **On So-called Soft Chancres.**

**NATIONAL HOSPITAL FOR THE PARALYSED AND EPILEPTIC, Queen Sq., Bloomsbury, W.C.**

**At 3.30 p.m.**

**Oct. 6,** Dr. James Collier, **Local Lesions of the Spinal Cord-Anatomy.**

### A Tourists' Handbook.

The P. and O. Pocket Book. Third issue. (London : A. and C. Black.)

The directors of the famous shipping company known all over the world as the "P. and O.," are determined to keep as much abreast of the times in the matter of literature for their clients as in all the other departments of their business. We say "literature" advisedly, for the Pocket Book before us is very unlike an ordinary guide-book in several respects. It contains chapters on various regions of the East, written by authors of established reputation for the particular subject allotted them. Thus Mrs. Steel writes on India, le Comte de Lesseps on the Suez Canal, Miss Grimshaw on the South Sea Islands, and authorities equally well known deal with other parts of the magic East. Then there are numerous maps and sixteen illustrations in colour, beautifully reproduced from the works of well-known artists. Withal, the volume is useful as well as interesting, for there is the fullest information on everything the passenger wants to be informed of. Tables of distances, coinages, trade returns, ensigns, and a hundred other matters are included, and there are also descriptions and plans of all the principal ports at which the P. and O. steamers call. Altogether the book makes one half inclined to go to sea just for the pleasure of ticking off the various points so carefully and lucidly detailed in it.

### AWARDS OF ENTRANCE SCHOLARSHIPS.

### ST. BARTHOLOMEW'S HOSPITAL.

*Senior Entrance Scholarships in Science.*—These (value £75 each) have been awarded to N. Mutch, B.A., of Emmanuel College, Cambridge, and A. F. S. Sladden, B.A., of Jesus College, Oxford.

### GUY'S HOSPITAL MEDICAL SCHOOL.

The following entrance scholarships and certificates have been awarded :—

*Senior Science Scholarship for University Students* (value £50).—Nathan Mutch, B.A., Emmanuel College, Cambridge. Certificate : H. W. Buber, B.A., Clare College, Cambridge.

*Junior Science Scholarships.*—(Value £150) J. F. G. Richards, Preliminary Scientific (M.B.) Class, Guy's Hospital; (value £60) W. L. Webb, Guy's Hospital. Certificate : G. S. Miller, Guy's Hospital.

*Entrance Scholarships in Arts.*—(Value £100) A. J. E. Smith, Rugby School; (value £50) H. W. Evans, Modern School, Bedford.

### ST. MARY'S HOSPITAL MEDICAL SCHOOL.

*Open Scholarships in Natural Science.*—(Value £145) B. W. Armstrong, Boston Grammar School; (value 75 guineas) F. P. Bennett, University College, Cardiff; (value 50 guineas) J. R. M. Whigham, Westminster City School; (value 50 guineas) F. C. Robbs, Clarence College, Gravesend.

*University Scholarships.*—(Value 60 guineas) A. W. Bourne, B.A., Downing College, Cambridge; (value 60 guineas) W. A. Berry, Queen's College, Belfast.

*Epsom College Scholarship.*—E. P. Langley.

### ANSWERS TO CORRESPONDENTS.

Cremation.—Last year there were thirteen crematoria in operation in Great Britain. The charges for cremation at Golder's Green are five guineas, exclusive of the chaplain's fee (10s. 6d.) and the purchase of an urn (10s. 6d. and upwards). The certificate of cremation is 2s. 7d. The charges at Woking are almost identical. Two medical certificates of the cause of death are required; one from the medical attendant during the last illness, and a confirmatory certificate from a specially qualified practitioner. All particulars will be given by the London Cremation Company, Ltd., 324 Regent Street, W.

"Live and Let Live."—We regret that we have not space for the publication of your letter, which deals with a subject already discussed very fully in our correspondence columns and elsewhere.

THE HOSPITAL October 3, 1908.

*Name* ....................................................

*Address* ....................................................

....................................................

This Coupon must accompany manuscript or contributions intended for The Hospital.

